# Supplementary material for: Molecularly distinct striatonigral neuron subtypes differentially regulate locomotion
Source: Nat Commun. 2025 Mar 19;16:2710. doi: 10.1038/s41467-025-58007-x (PMC11923167; doi:10.1038/s41467-025-58007-x)
Supplement: Supplementary file 1 — Supplementary Information [file 41467_2025_58007_MOESM1_ESM.pdf]

Title: Molecularly Distinct Striatonigral Neuron Subtypes Differentially Regulate Locomotion

Authors: Jie Dong[1], Lupeng Wang[1], Breanna T. Sullivan[1], Lixin Sun[1], Victor M. Martinez Smith[1], Lisa Chang[1], Jinhui Ding[2], Weidong Le[3,4], Charles R. Gerfen[5], and Huaibin Cai[1*]

Supplementary Figures and Figure Legends

Supplementary Fig. 1. Different expression pattern of *Tshz1* and *Kremen1* in the dorsal striatum

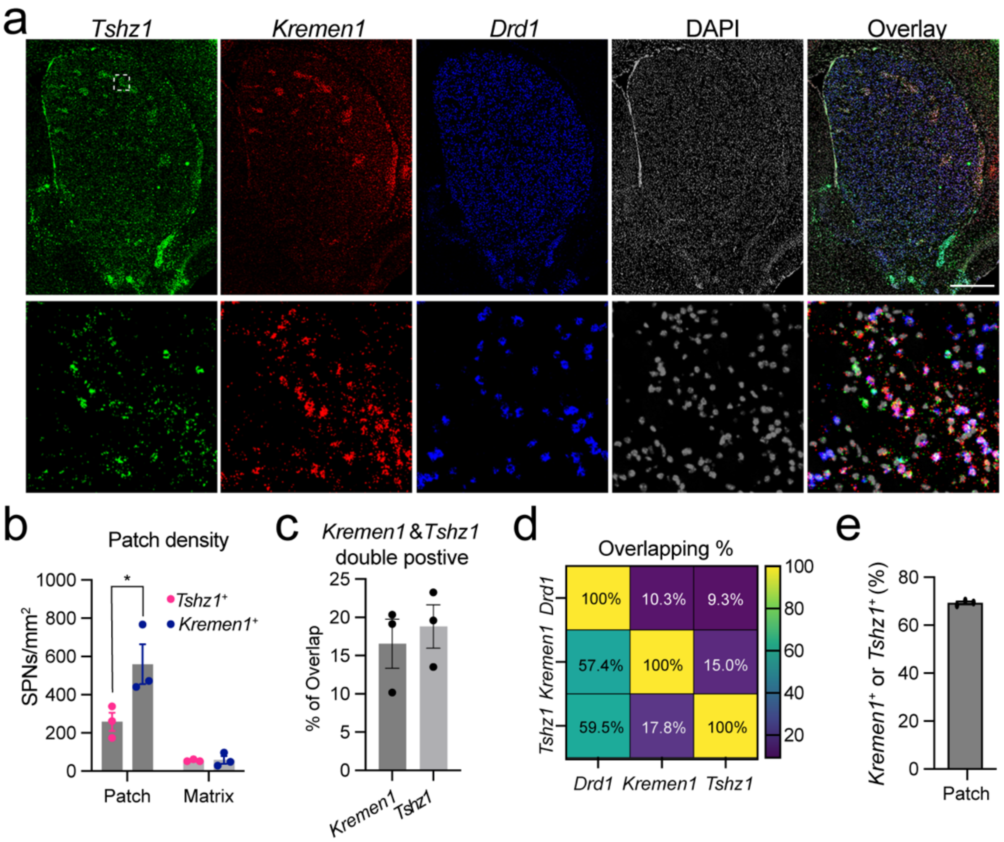

(**a**) Representative images showing RNAscope labeling for *Tshz1*, *Kremen1*, *Drd1* and DAPI in the striatum. Scale bar: 500 µm. High-magnification images of the boxed area in the top panel are shown at the bottom. (**b**) Comparison of *Tshz1*$^{+}$ SPN and *Kremen1*$^{+}$ SPN densities in the manually defined patch (*Tshz1*$^{+}$:259.0 ± 47.53 cells / mm^{2} vs. *Kremen1*$^{+}$:559.9 ± 103.9 cells / mm^{2}) and matrix (*Tshz1*$^{+}$:55.87 ± 3.53 cells / mm^{2} vs. *Kremen1*$^{+}$:58.39 ± 20.0 cells / mm^{2}) compartments in the dorsal striatum (n = 3 mice). Paired t-test, two-tailed, $^{*}p<0.05$. (**c**) Bar graph illustrating the percentages of double-positive dSPNs for *Kremen1* and *Tshz1* within individual *Kremen1*$^{+}$ and *Tshz1*$^{+}$ dSPNs. (**d**) Heatmap displaying the percentages of *Drd1*$^{+}$ nuclei positive for *Kremen1* and *Tshz1* (1st row), *Kremen1*$^{+}$ nuclei positive for *Drd1* and *Tshz1* (2nd row), and *Tshz1*$^{+}$ nuclei that were positive for *Drd1* and *Kremen1* (3rd row) (n = 3 mice). (**e**) Percentage of *Kremen1*$^{+}$ and *Tshz1*$^{+}$ SPNs in the manually defined patch compartment. All error bars represent mean ± SEM.

Supplementary Fig. 2. Generation of *Kremen1*$^{2A\text{-}Cre}$ knock-in mice

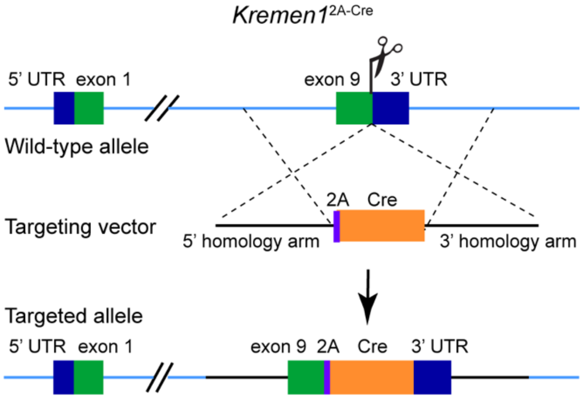

A schematic illustrating the insertion of the 2A-Cre coding sequence upstream of the stop codon in exon 9 of the *Kremen1* gene. A gene-targeting donor plasmid was constructed with a 3.2kb 5' homologous arm and a 2.9kb 3' homologous arm. The gRNA sequence (GTGGGCTTCAGTCACTCACG AGG) was used to guide the precise insertion of 2A-Cre DNA fragment via CRISPR/Cas9-mediated gene targeting.

Supplementary Fig. 3. Quantification of *Kremen1*$^{+}$ cells in the patch and matrix

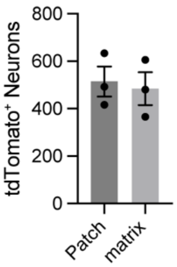

The average number of *Kremen1*$^{+}$ SPNs was quantified in the patch (positive for MOR1 immunostaining) and matrix compartments from two representative coronal hemisphere sections across three *Kremen1*$^{2A\text{-}Cre}$;Ai14 mice. *Kremen1*$^{+}$ SPNs were identified by tdTomato signals with a soma diameter larger than 10 µm. Paired t-test, two-tailed, $p = 0.6324$. All error bars represent mean ± SEM.

Supplementary Fig. 4. Identification of *Kremen1*$^{+}$ cell types in striatum

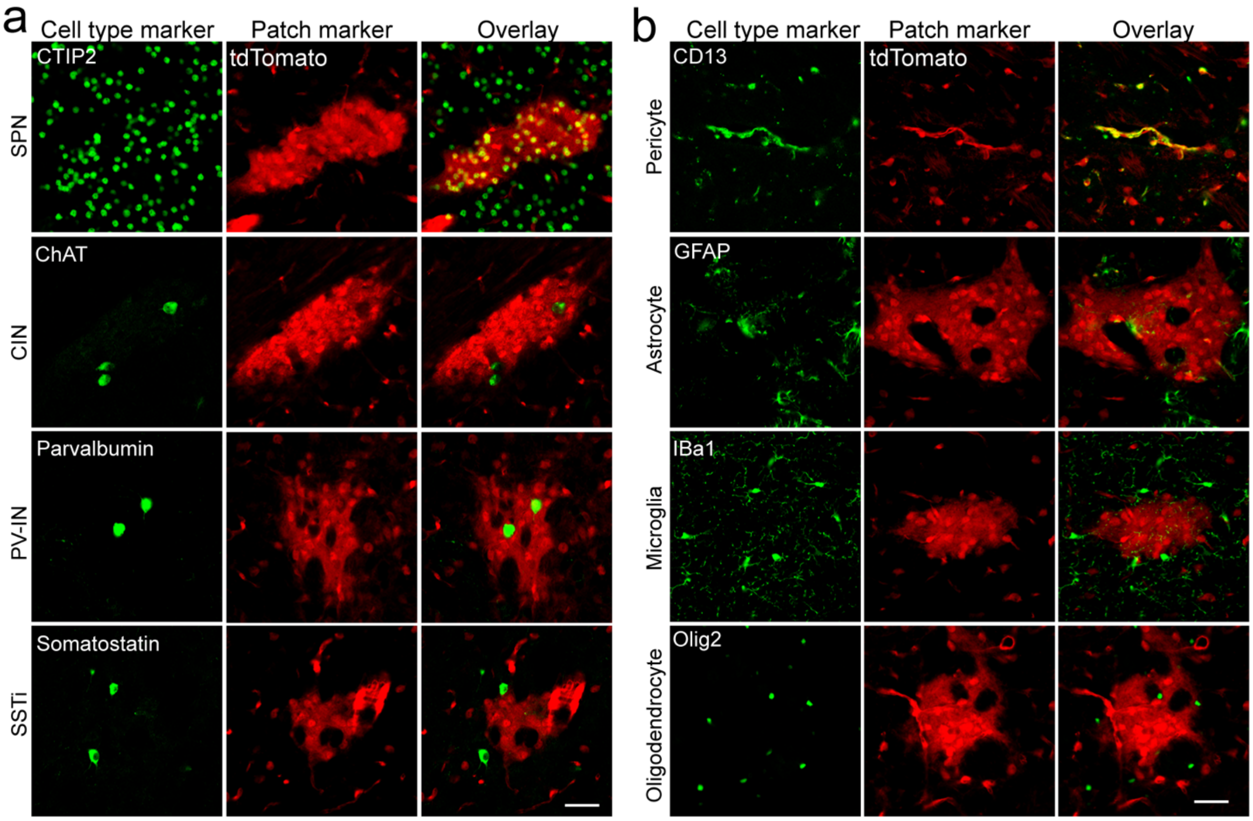

(**a**) Representative images of coronal sections from *Kremen1*$^{2A\text{-}Cre}$;Ai14 mice (n>3), co-stained with tdTomato and markers for various striatal neuronal types, including CTIP2 for SPNs, choline acetyltransferase (ChAT) for cholinergic interneurons (CINs), parvalbumin for parvalbumin interneurons (PVs), and somatostatin for somatostatin-expressing interneurons (SSTi). Scale bar: 50 µm. (**b**) Representative images of coronal sections from *Kremen1*$^{2A\text{-}Cre}$;Ai14 mice (n>3), co-stained with tdTomato and markers for glial and vascular cell types, including CD13 for pericytes, GFAP for astrocytes, IBA1 for microglia, and Olig2 for oligodendrocytes. Scale bar: 50 µm.

Supplementary Fig. 5. Characterization of the distribution and projection pattern of *Kremen1*⁺ neurons

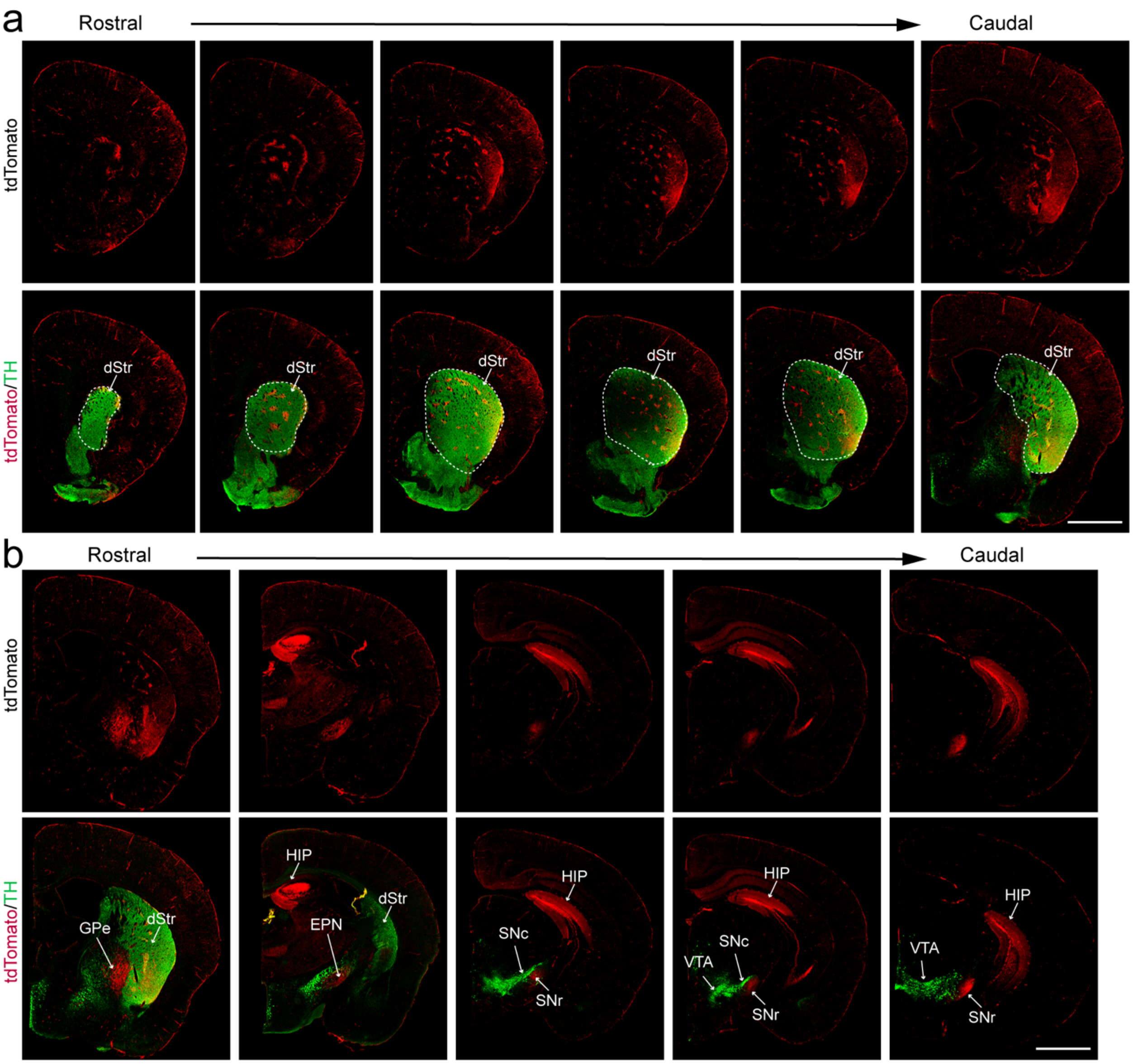

(**a**) Coronal sections from a representative *Kremen1*$^{2A\text{-}Cre}$;Ai14 mouse showing the *Kremen1* gene locus-directed tdTomato expression throughout the dorsal striatum. Scale bar: 1mm. (**b**) Coronal sections from a representative mouse showing the *Kremen1* gene locus-directed tdTomato expression in projection nucleuses, including GPe, ENP and SN. The tdTomato signals are also detected in the hippocampal (HIP) region. Scale bar: 1mm.

Supplementary Fig. 6. Characterization of the distribution and projection pattern of *Calb1*$^{+}$ SPNs

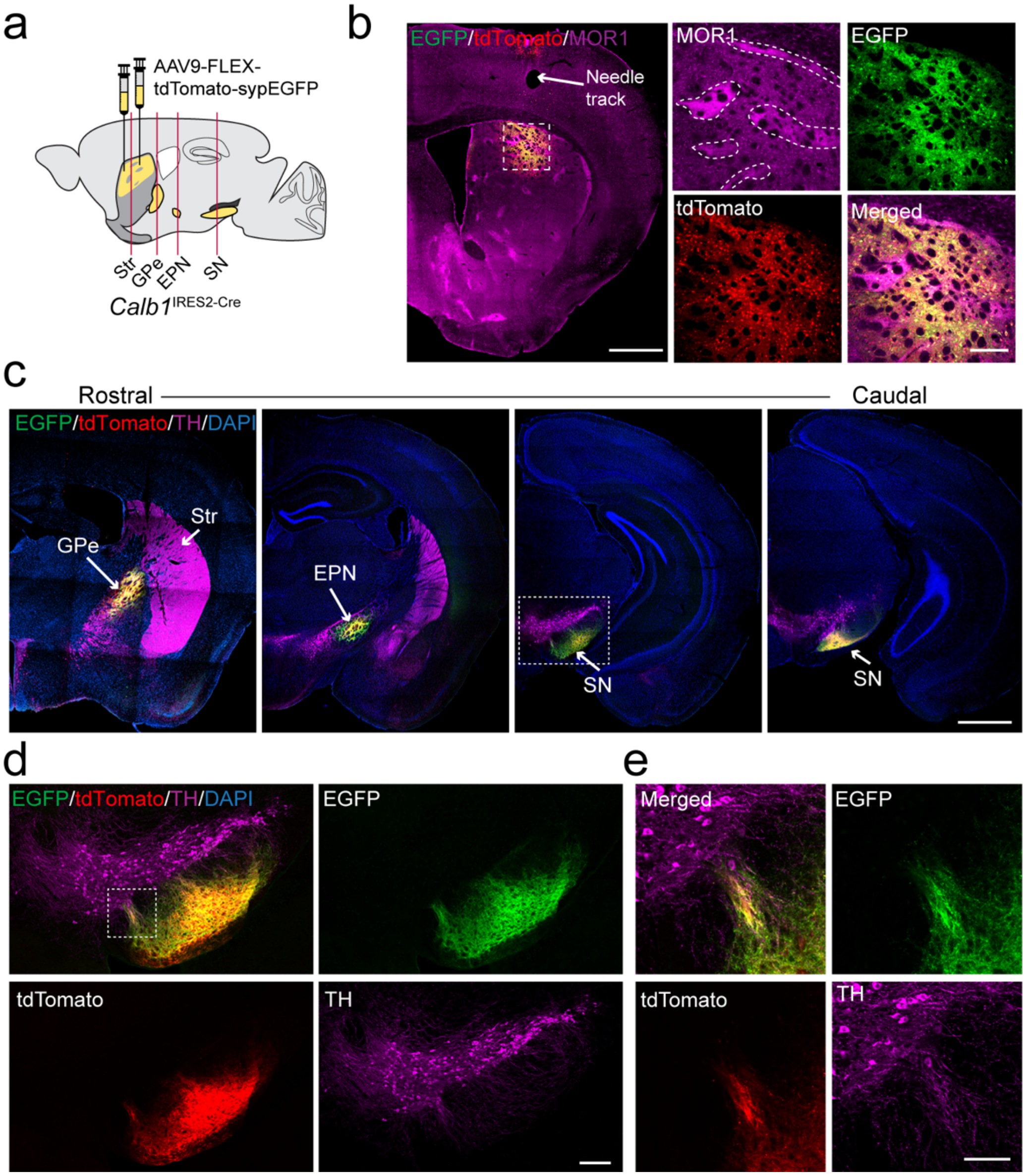

(**a**) Schematics of AAV9-FLEX-tdTomato-synEGFP injection in the dorsal striatum (dStr) of *Calb1*$^{IRES2\text{-}Cre}$ mice. The red vertical lines indicate the location of coronal sections shown in panels (**b)** and **(c)**. (**b**) Representative image of MOR1(purple), sypEGFP (green) and tdTomato (red) staining in the dStr. The boxed area in the left panel is magnified in the right panels. Scale bars: 500 µm in left panel and 100 µm in boxed area. (**c**) A series of coronal sections stained for TH (purple), sypEGFP (green), tdTomato (red) and DAPI (blue), illustrating the projections of *Calb1*$^{+}$ SPNs to globus pallidus externus (GPe), entopeduncular nucleus (EPN), and substantia nigra (SN). Scale bars: 500 mm. (**d**) Representative image of TH (purple), sypEGFP (green), tdTomato (red) staining in the SN. Scale bars: 200 µm. (**e**) The boxed area in (**d**) highlights the overlap between the axon terminals of *Calb1*$^{+}$ SPNs and the dendrites of TH^{+} DANs. Scale bar: 100 µm.

Supplementary Fig. 7. Validation of fiber photometry recording with GCaMP8s

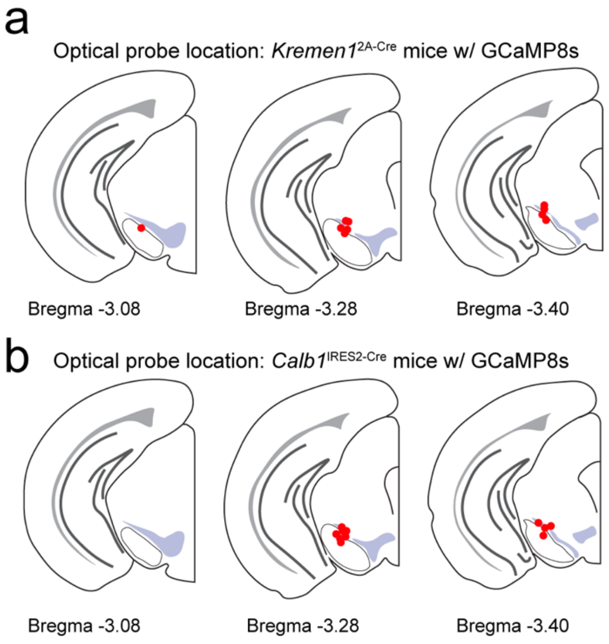

(**a, b**) Schematic diagrams showing fiber tip placements in the SN for *Kremen1*$^{2A\text{-}Cre}$ (**a**) and *Calb1*$^{IRES2\text{-}Cre}$ (**b**) mice.

Supplementary Fig. 8. Locomotion velocity comparison of *Kremen1*$^{2A\text{-}Cre}$ and *Calb1*$^{IRES2\text{-}Cre}$ mice

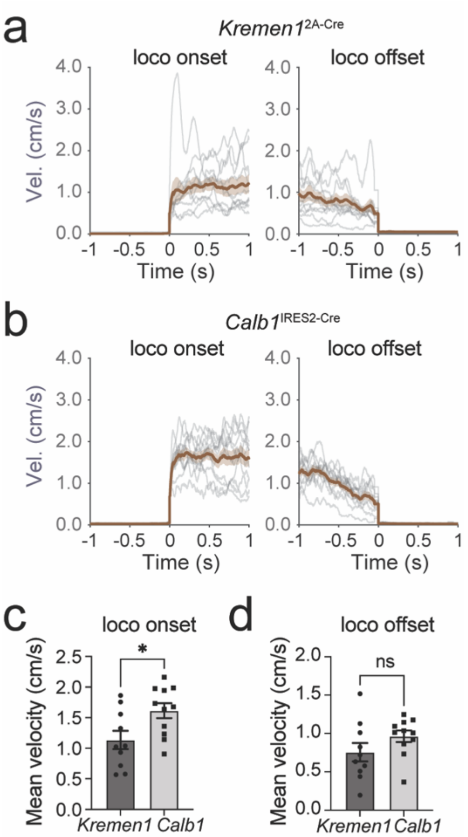

(**a**) Locomotion velocity traces of individual mice (gray) and population mean (brown) aligned on locomotion onset (left) and offset (right) of *Kremen1*$^{2A\text{-}Cre}$ (n = 10) mice. **b**) Locomotion velocity traces of individual mice (gray) and population mean (brown) aligned on locomotion onset (left) and offset (right) of *Calb1*$^{IRES2\text{-}Cre}$ (n = 11) mice. (**c**) Mean velocity comparison between *Kremen1*$^{2A\text{-}Cre}$ and *Calb1*$^{IRES2\text{-}Cre}$ mice during locomotion onset. Unpaired t-test, two tailed, $^{*}p = 0.022$. (**d**) Mean velocity comparison between *Kremen1*$^{2A\text{-}Cre}$ and *Calb1*$^{IRES2\text{-}Cre}$ mice during locomotion offset. Unpaired t-test, two tailed, p = 0.15.

Supplementary Fig. 9. Optogenetics activation of *Kremen1*$^{+}$ and *Calb1*$^{+}$ dSPNs at lower intensity or varying frequencies over a short period

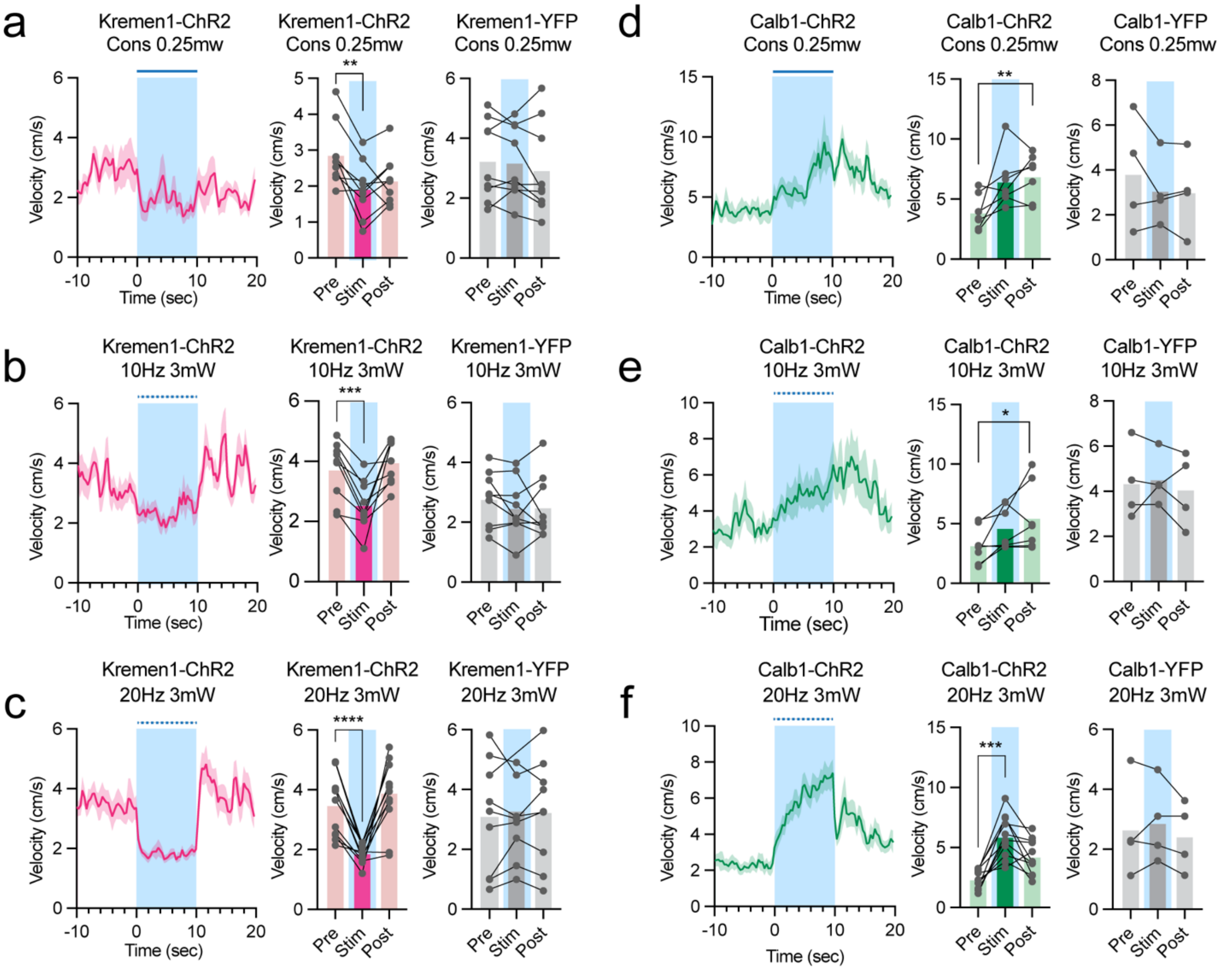

(**a-c**) Instantaneous velocity aligned to 0.25mW/constant (const, **a**), 3mW/10Hz (**b**), and 3mW/20Hz (**c**) light stimulations for 10s at the SN of Kremen1-ChR2 and Kremen1-YFP mice, respectively. (**a**) Kremen1-ChR2 mice, n = 9, repeated measures one-way ANOVA: F(1.888, 15.10) = 8.840, **p = 0.0032. Multiple comparisons test, Pre vs. Stim, **p = 0.0072. (**b**) Kremen1-ChR2 mice, n = 9, repeated measures one-way ANOVA: F(1.433, 11.47) = 11.45, **p = 0.0033. Multiple comparisons test, Pre vs. Stim, ***p = 0.0008; Stim vs. Post, *p = 0.0128. (**c**) Kremen1-ChR2 mice, n = 12, repeated measures one-way ANOVA: F(1.749, 19.23) = 25.33, ****p < 0.0001. Multiple comparisons test, Pre vs. Stim, ****p < 0.0001; Stim vs. Post, ***p = 0.0003. Optogenetics activation of Kremen1-YFP (n = 9) did not significantly change velocity. (**d-f**) Instantaneous velocity aligned to light stimulation at the SN of Calb1-ChR2 and Calb1-YFP with the same parameters as **a-c**. (**d**) Calb1-ChR2 mice, n = 7, repeated measures one-way ANOVA: F(1.325, 7.949) = 8.78, *p = 0.0143. Multiple comparisons test, Pre vs. Post, **p = 0.0091. (**e**) Calb1-ChR2 mice, n = 7, repeated measures one-way ANOVA: F(1.859, 11.15) = 6.565, *p = 0.0142. Multiple comparisons test, Pre vs. Post, *p = 0.03. (**f**) Calb1-ChR2 mice, n = 11, repeated measures one-way ANOVA: F(1.871, 18.71) = 24.08, ****p < 0.0001. Multiple comparisons test, Pre vs. Stim, ***p = 0.0002; Pre vs. Post, **p = 0.0038; Stim vs. Post, *p =

0.0335. Optogenetics activation of Calb1-YFP (n = 4) did not significantly change velocity. All error bars are represented as mean ± SEM.

Supplementary Fig. 10. Characterization of axon projections of *Kremen1*$^{+}$ iSPNs

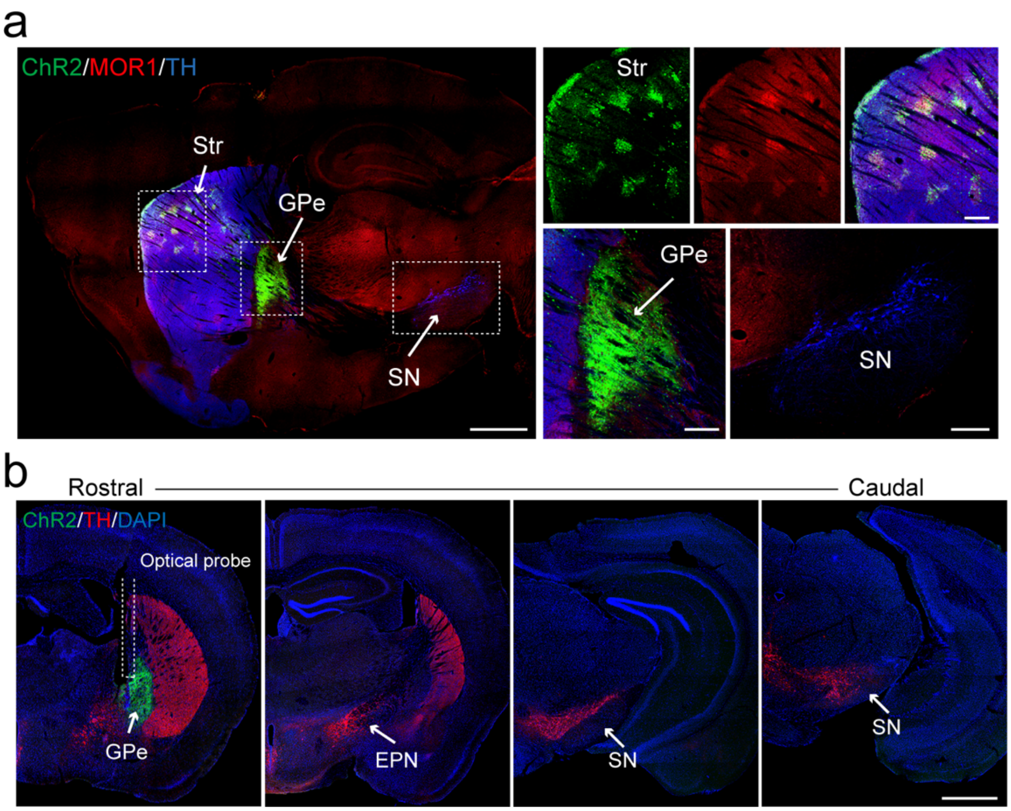

(**a**) Representative sagittal section from a *Kremen1*$^{2A\text{-}Cre}$;*A2a*$^{2A\text{-}Flp}$ double KI mouse injected with AAV1-ConFon-ChR2 in the dorsal striatum (Str), showing that ChR2 (green) expression is concentrated in MOR1 (red)-positive regions. Notably, *Kremen1*$^{+}$ iSPNs project exclusively to the GPe. The right panels show high-magnification images of the boxed areas in the left panel, highlighting the dorsal Str, GPe, and SNr. Scale bars: 1mm in whole sagittal image, 200 µm in boxed areas. (**b**) A series of coronal images showing ChR2 (green), TH (red), and DAPI (blue) in the GPe, EPN, and SNr. Scale bars: 500 µm.

Supplementary Fig. 11. Chemogenetic activation of *Kremen1*$^{+}$ SPNs suppresses locomotion

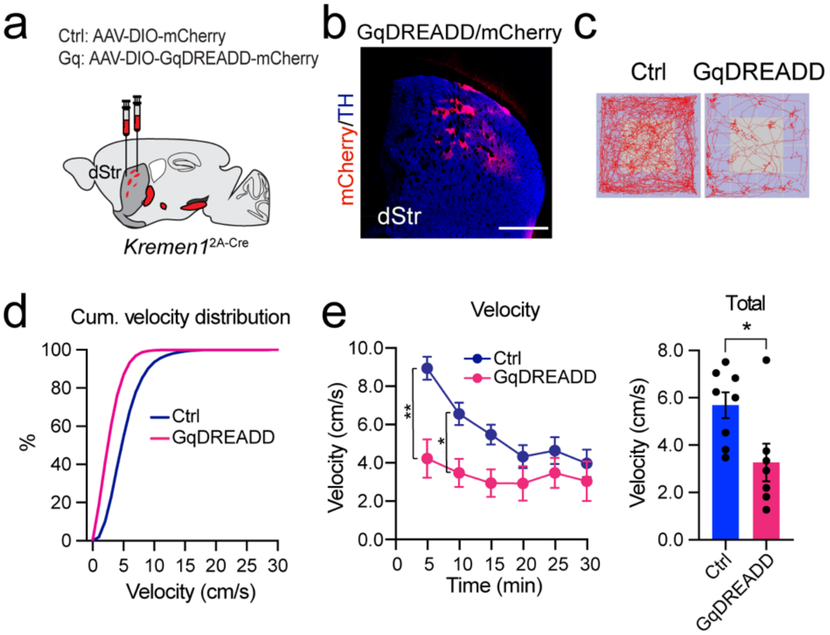

(**a**) Schematics illustrating the injection of Ctrl or GqDREADD AAV vectors in the dorsal striatum of *Kremen1*$^{2A\text{-}Cre}$ mice. (**b**) Representative image showing GqDREADD/mCherry expression in the patch-like structures in the dorsal striatum. Scale bar: 500 µm. (**c**) Tracks from representative Ctrl and GqDREADD mice during the open field test. (**d**) Cumulative (Cum.) frequency distribution of velocity in Ctrl (n=8 mice) and GqDREADD (n=7 mice) groups. (**e**) Velocity measurements: (left) velocity change over 5-minute intervals during a 30-minute test period and (right) the average velocity over the entire 30-minute test. In the left panel, two-way ANOVA shows a significant interaction (p = 0.0036) with post-hoc analysis indicating a significant difference at specific time points (*p = 0.035). In the right panel, the average velocity of the Ctrl group was 5.68 ± 0.55 cm/s, while the GqDREADD group showed a significantly reduced average velocity of 3.27 ± 0.79 cm/s (unpaired t-test, two-tailed, p = 0.0238). Data are presented as mean ± SEM.

Supplementary Fig. 12. Activation of *Kremen1*$^{+}$ and *Calb1*$^{+}$ dSPNs differentially modulates dopamine release during 10Hz optical stimulation

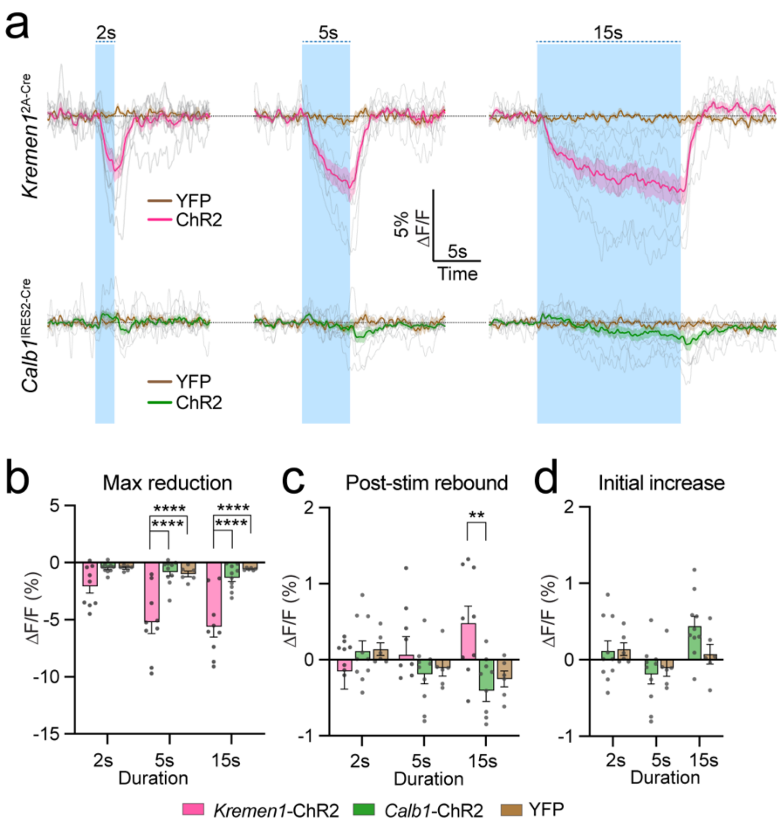

(**a**) Changes in dopamine levels (ΔF/F) in the dStr during 10 Hz light stimulation with varying durations in the SN of *Kremen1*$^{2A\text{-}Cre}$ (top) and *Calb1*$^{IRES2\text{-}Cre}$ (bottom) mice, along with YFP control mice. Solid traces with shading represent the population mean ± SEM of ΔF/F change for each group, aligned to the stimulation onset. Light gray traces show the mean ΔF/F change for individual *Kremen1* ChR2 (n = 9 mice) and *Calb1* ChR2 (n = 10 mice) mice. YFP data are from a combination of *Kremen1* YFP (n = 3 mice) and *Calb1* YFP (n = 3 mice). (**b**) The amplitude of maximum dopamine release reduction following activation of *Kremen1*$^{+}$ (red) and *Calb1*$^{+}$ (green) dSPN axons and their YFP (brown) controls, as shown in panel (**a**). Two-way ANOVA revealed an effect of dSPN activation ($F(2,66) = 38.18$, $^{****}p < 0.0001$), stimulation duration ($F(2,66) = 5.781$, $^{**}p = 0.0049$), and an interaction between the two factors ($F(4,66) = 3.006$, $*p = 0.0243$). Multiple comparisons showed significant differences between *Kremen1* ChR2, *Calb1*ChR2, and YFP groups (two-tailed, $^{****}p < 0.0001$). (**c**) Post-stimulation dopamine release changes after activation of *Kremen1*$^{+}$ and *Calb1*$^{+}$ dSPN axons, as shown in panel (**a**). Two-way ANOVA indicated an effect of dSPN activation ($F(2,66) = 2.338$, $p = 0.1045$), stimulation duration ($F(2,66) = 0.3171$, $p = 0.7294$), and an interaction effect ($F(4,66) = 3.350$, $^{*}p = 0.0147$). Multiple comparisons revealed significant differences in dopamine rebound amplitude between *Kremen1* ChR2, *Calb1* ChR2, and YFP groups (two-tailed, $^{**}p < 0.01$). (**d**) The amplitude of initial dopamine release elevation after stimulating *Calb1* ChR2 or YFP mice. Two-way ANOVA showed no significant effect of *Calb1*$^{+}$ dSPN activation ($F(1,42) = 0.6427$, $p = 0.4272$), but a significant effect of stimulation duration ($F(2,42) = 4.857$, $^{*}p = 0.0127$). No

significant differences were observed between *Calb1* ChR2 and YFP groups in the multiple comparison test. All data in this figure are presented as mean ± SEM.

Supplementary Fig. 13. Expression of Gabbr1 mRNA and protein in DANs and pericytes

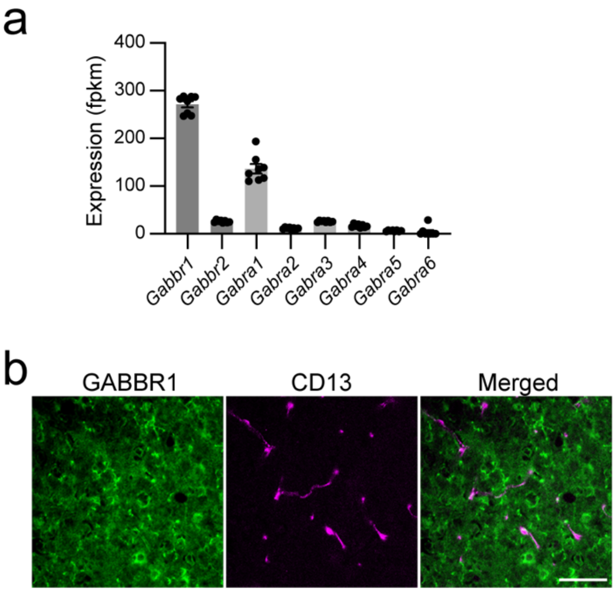

(**a**) Normalized expression of different GABA-B and GABA-A receptor encoding transcripts in the DANs isolated by laser capture microdissection from SNc (plotted from PRJNA775656). (**b**) Representative images showing GABBR1 receptor (green) and pericyte marker CD13 (magenta) staining in the SN of a Ctrl mouse. No detectable GABBR1 expression in the pericytes. Scale bar: 50 μm.

Supplementary Fig. 14. Expression of HA-tagged saCas9 in *Aldh1a1*[+] DANs

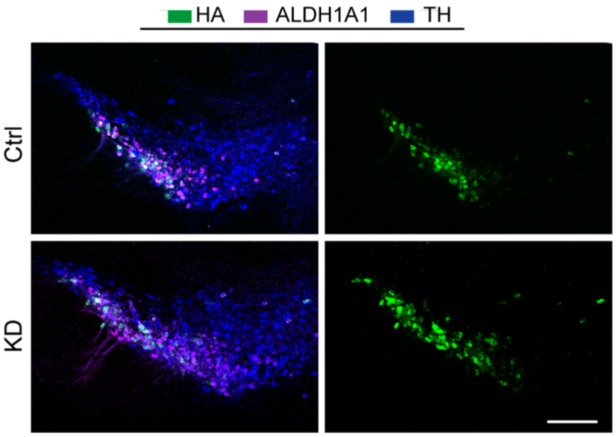

Representative image showing HA (green), ALDH1A1 (magenta), and TH (blue) stating in the SNc of Ctrl and *Gabbr1*-KD mice. Scale bar: 500 μm.

Supplementary Fig. 15. Cre-off ChR2 expression in the dorsal striatum

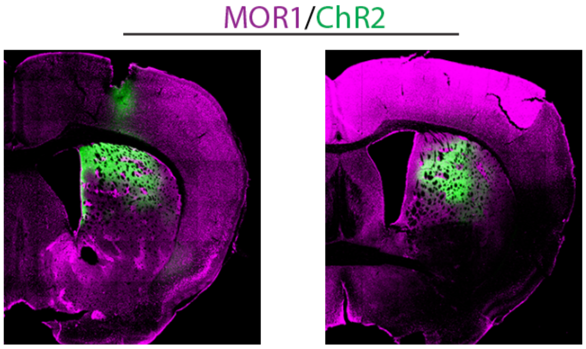

Two sequential images showing the expression of Cre-off ChR2 (green) in the matrix compartments, which are devoid MOR1 expression (magenta).

Supplementary Fig. 16. Individual traces of *Aldh1a1*$^+$ DAN activity during *Kremen1*$^+$ dSPN activation under *Gabbr1* Knockdown condition

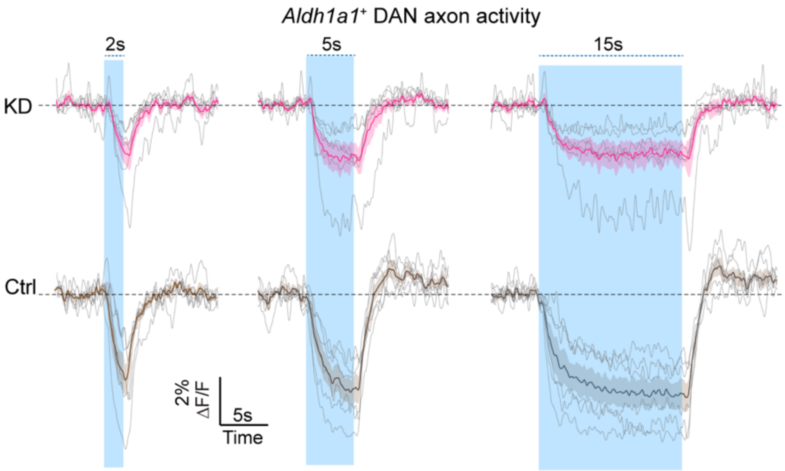

Individual traces of *Aldh1a1*$^+$ DAN axonal GCaMP8s activity in the dorsal striatum during 20 Hz light stimulation of varying durations (adapted from **Fig. 10c**). Traces from Gabbr1 knockdown (KD) mice (N = 6, top) and control (Ctrl) mice (N = 6, bottom) are shown in light gray. Group-averaged responses (solid line) ± SEM (shaded area) provide context.
