# Supplementary material for: Molecularly distinct striatonigral neuron subtypes differentially regulate locomotion
Source: Nat Commun. 2025 Mar 19;16:2710. doi: 10.1038/s41467-025-58007-x (PMC11923167; doi:10.1038/s41467-025-58007-x)
Supplement: Supplementary file 2 — Reporting Summary [file 41467_2025_58007_MOESM2_ESM.pdf]

nature portfolio

Corresponding author(s): Huaibin Cai

Last updated by author(s): Feb 3, 2025

Reporting Summary

Nature Portfolio wishes to improve the reproducibility of the work that we publish. This form provides structure for consistency and transparency in reporting. For further information on Nature Portfolio policies, see our Editorial Policies and the Editorial Policy Checklist.

Statistics

For all statistical analyses, confirm that the following items are present in the figure legend, table legend, main text, or Methods section.

n/a	Confirmed	
☐	☒	The exact sample size (*n*) for each experimental group/condition, given as a discrete number and unit of measurement
☐	☒	A statement on whether measurements were taken from distinct samples or whether the same sample was measured repeatedly
☐	☒	The statistical test(s) used AND whether they are one- or two-sided<br>*Only common tests should be described solely by name; describe more complex techniques in the Methods section.*
☒	☐	A description of all covariates tested
☒	☐	A description of any assumptions or corrections, such as tests of normality and adjustment for multiple comparisons
☐	☒	A full description of the statistical parameters including central tendency (e.g. means) or other basic estimates (e.g. regression coefficient) AND variation (e.g. standard deviation) or associated estimates of uncertainty (e.g. confidence intervals)
☐	☒	For null hypothesis testing, the test statistic (e.g. *F*, *t*, *r*) with confidence intervals, effect sizes, degrees of freedom and *P* value noted<br>*Give P values as exact values whenever suitable.*
☒	☐	For Bayesian analysis, information on the choice of priors and Markov chain Monte Carlo settings
☒	☐	For hierarchical and complex designs, identification of the appropriate level for tests and full reporting of outcomes
☒	☐	Estimates of effect sizes (e.g. Cohen's *d*, Pearson's *r*), indicating how they were calculated

Our web collection on statistics for biologists contains articles on many of the points above.

Software and code

Policy information about availability of computer code

Data collection	1. Synapse (Tucker-Davis Technologies) was used to record GCaMP8s, DA3m fiber photometry signals.<br>2. Laser scanning confocal microscope LSM 780 (Zeiss) with Zen software were used for fluorescent images acquirement.<br>3. For RNA-sequencing, the libraries were qualified using the Bioanalyzer DNA 1000 assay (Agilent) and sequenced with Illumina HiSeq 2000. The standard Illumina pipeline was used to generate Fastq files.
Data analysis	1. EthoVision XT software (Noldus) was used to track the mice and analyze the video for velocity, time, distance travelled and movement bouts.<br>2. Imaris (v10.0.0, Bitplane, Belfast Northern Ireland, UK) was used for RNAscope images analysis .<br>3. ImageJ (v1.53t) was used for immunostaining analysis.<br>4. MATLAB (MathWorks, R2023_a) was used for fiber photometry signals process and analysis. The code that supports the findings of this study is available upon publication.<br>5. Figures were plotted using Prism Graphpad 9.0 or MATLAB (MathWorks, R2023_a).

For manuscripts utilizing custom algorithms or software that are central to the research but not yet described in published literature, software must be made available to editors and reviewers. We strongly encourage code deposition in a community repository (e.g. GitHub). See the Nature Portfolio guidelines for submitting code & software for further information.

Data

Policy information about availability of data

All manuscripts must include a data availability statement. This statement should provide the following information, where applicable:

- Accession codes, unique identifiers, or web links for publicly available datasets
- A description of any restrictions on data availability
- For clinical datasets or third party data, please ensure that the statement adheres to our policy

Data Availability
The accession number of the striatal tissue RNA-seq data is PRJNA870469. The accession number of SNc RNA-seq data is PRJNA775656. All data generated or analyzed during this study are included in this published article. Source data are provided with this paper.

Code Availability
The code used to process the imaging data in this study has been deposited in GitHub (https://github.com/jdNIH/Kremen1_2025).

Research involving human participants, their data, or biological material

Policy information about studies with human participants or human data. See also policy information about sex, gender (identity/presentation), and sexual orientation and race, ethnicity and racism.

Reporting on sex and gender	N/A
Reporting on race, ethnicity, or other socially relevant groupings	N/A
Population characteristics	N/A
Recruitment	N/A
Ethics oversight	N/A

Note that full information on the approval of the study protocol must also be provided in the manuscript.

Field-specific reporting

Please select the one below that is the best fit for your research. If you are not sure, read the appropriate sections before making your selection.

☒ Life sciences ☐ Behavioural & social sciences ☐ Ecological, evolutionary & environmental sciences

For a reference copy of the document with all sections, see nature.com/documents/nr-reporting-summary-flat.pdf

Life sciences study design

All studies must disclose on these points even when the disclosure is negative.

Sample size	Sample sizes were not predetermined before experiment, but the sample sizes for experiments was determined to provide enough statistical power based on power analysis of preliminary data. Samples size for each experiment is described in the Figure legend.
Data exclusions	No data were excluded from the analysis.
Replication	1. RNAscope quantification was performed independently by two different investigators. The results were consistent between different investigators.<br>2. Fiber photometry GCaMP8s recording was consistent with our preliminary experiment which we used GCaMP6s-axon.<br>3. Optogenetics behavioral data and fiber photometry recordings were repeated several times with at least two different cohorts of animals. The replication were consistent across different cohorts.<br>4. For the fluorescent images as representative panels in the figures, no less than 3 mice from the same treatment group were checked for the validity.
Randomization	The samples for fiber photometry recording and behavioral experiments were grouped based on age and gender balance. Within each group, the selection of individual animal was totally randomized.
Blinding	The investigators were blinded to groups when carrying out the fiber photometry and behavioral experiments.

Reporting for specific materials, systems and methods

We require information from authors about some types of materials, experimental systems and methods used in many studies. Here, indicate whether each material, system or method listed is relevant to your study. If you are not sure if a list item applies to your research, read the appropriate section before selecting a response.

Materials & experimental systems

n/a	Involved in the study
☐	☒ Antibodies
☒	☐ Eukaryotic cell lines
☒	☐ Palaeontology and archaeology
☐	☒ Animals and other organisms
☒	☐ Clinical data
☒	☐ Dual use research of concern
☒	☐ Plants

Methods

n/a	Involved in the study
☒	☐ ChIP-seq
☒	☐ Flow cytometry
☒	☐ MRI-based neuroimaging

Antibodies

Antibodies used	The primary antibodies used for immunostaining included rabbit monoclonal anti-TH (Pel-Freez Biologicals, P40101; dilution 1:1000), mouse monoclonal anti-TH (ImmunoStar, 22941; dilution 1:1000), chicken polyclonal anti-TH (Aves Labs, TYH; dilution 1:500), rabbit anti-Mu-opioid receptor (MOR) (ImmunoStar, 24216; dilution 1:3000), chicken polyclonal anti-GFP (Aves Labs, GFP-1020; dilution 1:1000), mouse polyclonal anti-RFP (Rockland, 200-301-379; dilution 1:1000), guinea pig anti-Parvalbumin (Swant, GP72; dilution 1:1000), rabbit anti-Somatostatin-14 (T-4103, Peninsula Laboratories; dilution 1:1000), goat anti-ChAT (AB144P, Millipore; dilution 1:500), rabbit anti-Iba1 (Wako, 019-19741, 1:1000), rabbit anti-GFAP (Abcam, ab7260; dilution 1:2000), goat anti-CD13 (R&D Systems, AF2335; dilution 1:100), and mouse monoclonal anti-GABAB (Abcam, ab55051, dilution 1:500). Appropriate fluorophore-conjugated secondary antibodies (Life Technologies) were used depending on the desired fluorescence colors.
Validation	The specificity of antibodies was verified through comparing with previous published studies and manufacturer's website.

Animals and other research organisms

Policy information about studies involving animals; ARRIVE guidelines recommended for reporting animal research, and Sex and Gender in Research

Laboratory animals	The Kremen12A-Cre KI mice were generated by Shanghai Model Organisms Inc. (Shanghai, China). The Aldh1a1CreERT2 KI mice were generated as previously described in Wu, et al, Cell Reports, 2019. The Nr4a1–eGFP (Stock No: 036737-UCD) transgenic mice were obtained from Mutant Mouse Resource & Research Centers (MMRRC). The Calb1IRES2-Cre mice (Stock No: 028523) and Ai14 (Stock No: 007908) were obtained from the Jackson laboratory. The A2aFlp KI mice were generated by the Rodent Transgenic Core of National Institute of Mental Health (NIMH). Both females and males were used for all experiments. Mice used for viral injections and behavior were between 2 and 4 months of age.
Wild animals	N/A
Reporting on sex	Both females and males were used for all experiments, and no differences between sexes were found.
Field-collected samples	N/A
Ethics oversight	All mouse studies were conducted in accordance with the guidelines approved by the Institutional Animal Care and Use Committees (IACUC) of the Intramural Research Program of the National Institute on Aging (NIA), NIH.

Note that full information on the approval of the study protocol must also be provided in the manuscript.

Plants

Seed stocks	N/A
Novel plant genotypes	N/A
Authentication	N/A
